# Diverse lattice dynamics in ternary Cu-Sb-Se compounds

**DOI:** 10.1038/srep13643

**Published:** 2015-09-02

**Authors:** Wujie Qiu, Lihua Wu, Xuezhi Ke, Jihui Yang, Wenqing Zhang

**Affiliations:** 1 Institute of Theoretical Physics and Department of Physics, East China Normal University, Shanghai 200241, China; 2 State Key Laboratory of High Performance Ceramics and Superfine Microstructure, Shanghai Institute of Ceramics, Chinese Academy of Sciences, Shanghai 200050, China; 3 Materials Science and Engineering Department, University of Washington, Seattle, Washington 98195, USA; 4 Materials Genome Institute, Shanghai University, Shanghai 200444, China

## Abstract

Searching and designing materials with extremely low lattice thermal conductivity (LTC) has attracted considerable attention in material sciences. Here we systematically demonstrate the diverse lattice dynamics of the ternary Cu-Sb-Se compounds due to the different chemical-bond environments. For Cu_3_SbSe_4_ and CuSbSe_2_, the chemical bond strength is nearly equally distributed in crystalline bulk, and all the atoms are constrained to be around their equilibrium positions. Their thermal transport behaviors are well interpreted by the perturbative phonon-phonon interactions. While for Cu_3_SbSe_3_ with obvious chemical-bond hierarchy, one type of atoms is weakly bonded with surrounding atoms, which leads the structure to the part-crystalline state. The part-crystalline state makes a great contribution to the reduction of thermal conductivity that can only be effectively described by including a rattling-like scattering process in addition to the perturbative method. Current results may inspire new approaches to designing materials with low lattice thermal conductivities for high-performance thermoelectric conversion and thermal barrier coatings.

Designing novel and efficient energy-conversion materials has been of great importance in extricating the predicaments of global energy shortage and underutilization of energy resources. Thermoelectric (TE) materials are capable of converting waste heat directly into electricity^1^. The performance of a TE material is governed by the dimensionless figure of merit, defined as 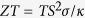, where *T* is the absolute temperature, *S* the Seebeck coefficient, *σ* the electrical conductivity, and *κ* the thermal conductivity. The *κ* includes the lattice and the electronic components. One effective way to improve *ZT* is to reduce the *κ*, especially the lattice thermal conductivity (LTC), via enhancing phonon scattering rates^2^,^3^,^4^,^5^,^6^,^7^. In our previous study^8^, a concept of part-crystalline part-liquid (PCPL) state was proposed. Materials in such a state, manifesting the coexistence of rigid crystalline sublattices and fluctuating noncrystalline substructures, are very likely to possess extremely low LTCs, which can be treated as potentially promising TE materials.

Investigating intrinsic crystal structures and corresponding lattice dynamic properties is conducive to understanding the differences between crystalline and PCPL materials. Ternary Cu-Sb-Se materials (Cu_3_SbSe_4_, CuSbSe_2_, and Cu_3_SbSe_3_) provide a suitable platform, as they are composed by the same elements but present distinct crystal structures, and consequently dissimilar thermal transport properties^9^. Their intrinsically low LTCs are gratifying for TE conversion, especially the extremely low LTC in Cu_3_SbSe_3_^10^,^11^. The LTCs of Cu_3_SbSe_4_ and CuSbSe_2_ show a classical temperature dependence of *T*^−1^, while that of Cu_3_SbSe_3_ is nearly temperature-independent^10^. Such an abnormal thermal transport behavior has also been found in many other material systems, such as Cu_2_Se^12^,^13^, AgSbTe_2_^14^,^15^,^16^ and *β*-K_2_Bi_8_Se_13_^17^. Different interpretations have been proposed to qualitatively explain these anomalous low intrinsic LTC, including strong anharmonicity^18^,^19^, lone *s*^2^ pair interaction^9^,^14^,^15^, and complex compositions^17^,^20^. However, the physical origin of the low LTC is still a riddle in respect of lattice dynamics. Based on *ab initio* density functional calculations, here we systematically study the harmonic and anharmonic temperature-dependent lattice dynamics of the three Cu-Sb-Se compounds. The results indicate that compounds with and without chemical-bond hierarchy will display quite diverse behaviors in atomic vibrations, phonon-phonon interactions, and thermal transports, which can provide us an effective strategy to search and design TE materials with low LTCs.

## Results and Discussion

### Crystal structures and dynamic behaviors

**Figure 1:**
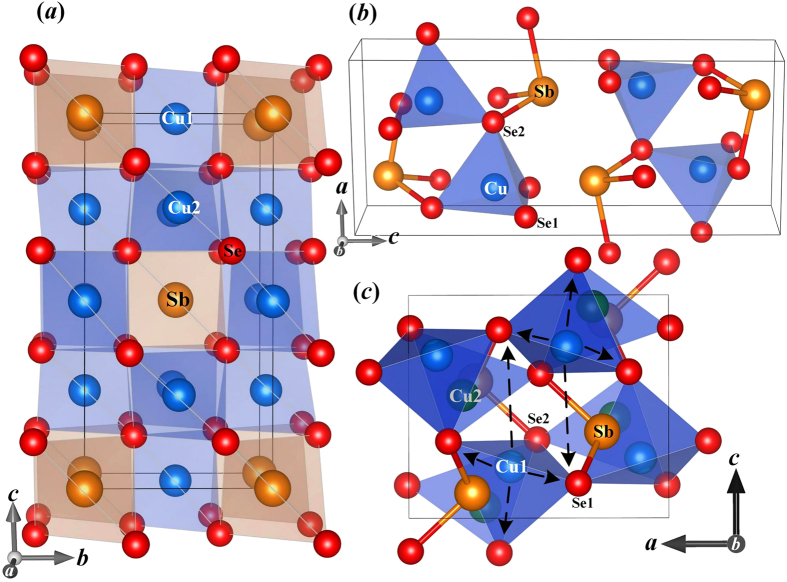
Crystal structures (a) Cu_3_SbSe_4_. (b) CuSbSe_2_. (c) Cu_3_SbSe_3_.

The calculated equilibrium lattice constants for Cu_3_SbSe_4_ (*a* = *b* = 5.736 Å and *c* = 11.411 Å), CuSbSe_2_ (*a* = 6.467 Å, *b* = 4.045 Å, and *c* = 15.048 Å) and Cu_3_SbSe_3_ (*a* = 8.099 Å, *b* = 10.672 Å, and *c* = 6.936 Å) are in good agreement with the experimental data^21^,^22^ and a previous study^10^. The compounds Cu_3_SbSe_4_ and CuSbSe_2_ have the diamond-like structures, as shown in Fig. 1(a,b), with Cu atoms occupied in the center of Se-formed tetrahedrons. As shown in Fig. 1(c), Cu_3_SbSe_3_ has an orthorhombic crystal structure with the *Pnma* space group. The Cu atoms mainly locate in the Se-formed tetrahedrons; however, the intrinsic structural channel allows Cu1 atoms to vibrate with large amplitudes around their equilibrium positions, especially in the *z* direction. The key structural difference between Cu_3_SbSe_4_ and CuSbSe_2_ is that the Sb atoms are whether disengaged from the tetrahedrons and the crystal structure is whether twisted due to the lone pair *s*^2^-induced redistribution in the former compound. Though the sublattices of Cu atoms look similar among the three compounds, there is no intrinsic structural channel in Cu_3_SbSe_4_ or CuSbSe_2_.

**Figure 2:**
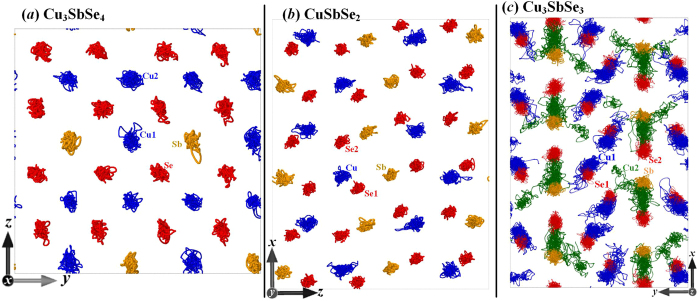
Trajectories of atoms from molecular dynamics simulations for (a) Cu_3_SbSe_4_, (b) CuSbSe_2_, and (c) Cu_3_SbSe_3_ at 400 K.

Figure 2 shows the trajectories of atoms from MD simulations at 400 K. For Cu_3_SbSe_4_ and CuSbSe_2_, both Fig. 2(a,b) illustrate that all the atoms are constrained around their equilibrium positions, indicating that they are in the crystalline state. However for Cu_3_SbSe_3_ (Fig. 2(c)), the part-liquid sublattice appears from the liquid-like random diffusion of Cu atoms, whereas the Se and Sb atoms are constrained around their equilibrium positions. The compound is thus in a mixed part-crystalline part-liquid state, containing one crystalline rigid part and the other liquid fluctuating sublattice^8^. To gain a better understanding about the origin of differentiated dynamic behaviors in these ternary compounds, the chemical-bond strength should be investigated.

**Figure 3:**
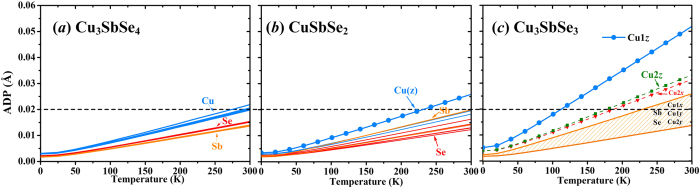
Calculated atomic displacement parameters (ADP) for three compounds. For (**c**) Cu_3_SbSe_3_, the ADPs for Sb, Se, Cu2*y*, Cu1*x*, and Cu1*y* are within the belt region. The dashed line is for a guide for the eye.

Atomic displacement parameter (ADP), which is defined as the mean-square amplitude of vibration of an atom around its equilibrium position, is calculated based on full phonon dispersions^8^,^23^. A relatively larger ADP value generally means that the corresponding atom vibrates more about its equilibrium position than other atoms, physically implicating the weak restoring forces on the vibrating atoms due to the existence of the weak bonding^24^,^25^. The ADPs of the atoms in Cu_3_SbSe_4_ and CuSbSe_2_ are almost in the homogenous level (<0.02 Å^2^), as shown in Fig. 3(a,b), respectively. None of the atoms in the two compounds is relatively weakly bonded, and consequently their melting points should be comparable due to the nonhierarchical chemical bonds, according to the classical Lindemann criterion of melting^26^. However, as shown in Fig. 3(c), the calculated ADP data of Cu1 atoms in the *z* direction (Cu1*z*) is at least twice larger than those for other species in Cu_3_SbSe_3_. The Cu1*z* accordingly are weakly bonded, and thus the compound reveals bonding strength hierarchy and atomic-level inhomogeneity. The melting state firstly occurs in the Cu1*z*-participated sublattice as the temperature increases. Similar behaviors can be observed in the Cu2 atoms due to their large ADP values along the *x* and *z* directions. In this regard, the appearance of the mixed PCPL state in Cu_3_SbSe_3_, as also represented in Fig. 2(c), is ascribed to the bonding strength hierarchy. Indeed, the experimental melting points of Cu_3_SbSe_4_ and CuSbSe_2_ are about 730 K^27^ and 750 K^28^, respectively, which demonstrates the typical crystalline character and homogeneous bonding strength in the two systems. Furthermore, the order-disorder transition of Cu atoms in Cu_3_SbSe_3_ was observed at finite temperatures^29^,^30^. Bonding strength hierarchy thus becomes an indicator of the appearance of part-crystalline state.

### Harmonic and anharmonic properties

Accordingly, investigating both harmonic and anharmonic phonon interactions at low temperatures can render deep understandings for different types of Cu-Sb-Se compounds. In the model adopted for calculating the LTC, three physical parameters (Debye temperatures, group velocities, and Grüneisen parameters) should be determined from several theoretical approximations, and the details are represented in Refs 8,10. For the three compounds, these parameters for each acoustic phonon mode were averaged by the weight of high-symmetry points, which are listed in Table 1. The CuSbSe_2_ compound is a “transitional” structure, since it possesses the homogeneous bonding strength (or ADP values) similar to Cu_3_SbSe_4_ and the lone pair *s*^2^ electrons similar to Cu_3_SbSe_3_. Harmonic properties, which are the reflections of the bonding stiffness including Debye temperatures and group velocities, of CuSbSe_2_ correspondingly lie between those of Cu_3_SbSe_4_ and Cu_3_SbSe_3_, as shown in Table 1.

**Figure 4:**
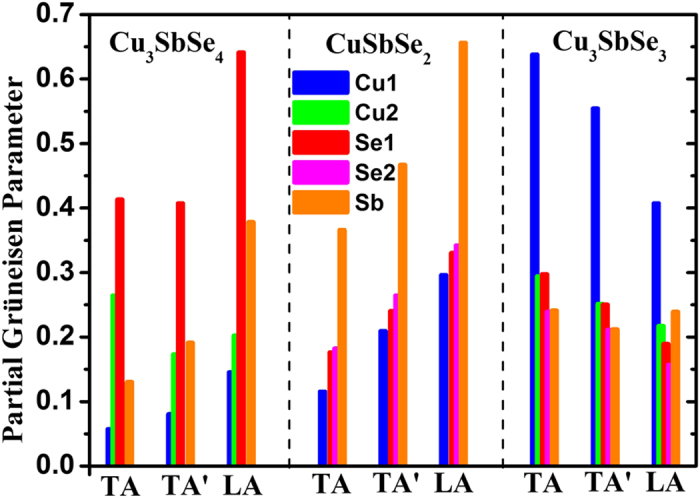
Average partial Grüneisen parameters for the TA, TA’, and LA mode for three compounds.

The anharmonic properties, which cannot be characterized by either atomic trajectories or ADPs, are represented by the Grüneisen parameter (*γ*), which is related to the third-order (or even higher-order) anharmonic potential well. The intensity of phonon anharmonicity of CuSbSe_2_ also lies between those of Cu_3_SbSe_4_ and Cu_3_SbSe_3_ (Table 1). To clarify the origins of phonon anharmonic interactions in Cu-Sb-Se compounds at low temperatures, partial Grüneisen parameters, describing the projected contributions from given atom types, are estimated by projecting the total Grüneisen parameter *γ*(**q**, *i*) onto an atom type *μ* in the *α* direction as follows^8^,


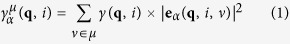


where **e**_*α*_(**q**, *i*, *v*) is the phonon polarization vector of a set of atoms *ν* derived from the dynamical matrix, *i* the phonon mode, and **q** the wave vector. The averaged partial Grüneisen parameters for transverse acoustic (TA with a lower group velocity and TA’ with a higher one) and longitudinal acoustic (LA) modes are calculated by 
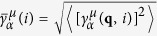
, which are shown in Fig. 4. For the close-packed diamond-like Cu_3_SbSe_4_ structure, the phonon anharmonic interactions is closely associated with the Se atoms (Fig. 4), which form 3D-frameworks for the whole system based on the CuSe_4_ and SbSe_4_ tetrahedrons. In this quasi-isotropic rigid network, the phonon-phonon interactions consequently are mainly determined by the spatial topology of Se atoms. However, as shown in Fig. 4, the averaged Grüneisen parameters of CuSbSe_2_ are mainly contributed by the Sb^3+^ ions with lone pair *s*^2^ electrons, since the residual non-bonding electrons begin to affect phonon modes through producing an extra electrostatic repulsion^9^,^14^,^15^. While for the Cu_3_SbSe_4_ and CuSbSe_2_, the appearance of the PCPL state can be excluded from the localized atomic trajectories in the MD simulations and the homogenous ADP values. The intrinsic structure characteristic therefore becomes a key in determining the lattice anharmonicity and phonon-phonon interactions. For the Cu_3_SbSe_3_, though the validity of the concept of phonon becomes questionable due to the diffusive behavior in the part-crystalline part-liquid state, quasi phonons or even localized vibrations can still exist at very low temperatures. As confirmed in the previous study^8^, the partial Grüneisen parameters of the Cu1 atoms are at least twice larger than those of other atoms for acoustic phonons, indicating that the Cu1 atoms play a predominant role in the intrinsic phonon-phonon interactions at low temperatures. Additionally, the Grüneisen parameters for the Cu1 atoms are overwhelmingly contributed by the *z* component^8^, which is consistent with the abnormal large Cu1*z* ADP and the liquid-like fluctuation behavior at finite temperatures.

### Lattice thermal conductivity

**Figure 5:**
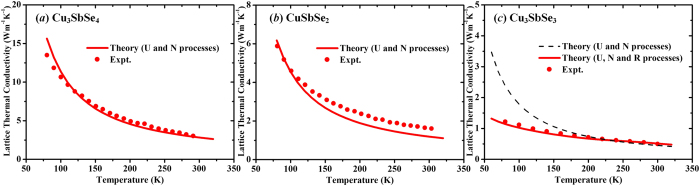
Temperature-dependent lattice thermal conductivity for the three compounds. The dotted lines refer to the experimental data. The solid and dashed lines are our calculated results.

By using the harmonic and anharmonic parameters given in Table 1 and the modified Debye-Callaway model, the LTCs can be estimated for the three compounds. Based on the aforementioned analysis, it should be rational to treat bond-homogenous Cu_3_SbSe_4_ and CuSbSe_2_ compounds as traditional crystalline solids, and the corresponding thermal transport behaviors are expected to be described by the classical perturbation techniques. As shown in Fig. 5(a,b), the theoretical temperature dependences (~*T*^−1^) show acceptable agreements with the experimental data^9^ by only considering the U and N processes for total phonon scattering rates. The slightly overestimation of the phonon-phonon interaction strength for the CuSbSe_2_ compound should be attributed to the inaccurate estimates by using three-phonon processes for the higher-order nonlinear correction caused by the lone pair *s*^2^ electrons^31^. Generally, the well-fitted temperature dependence indicates that the anharmonic effect of the extra electrostatic repulsion given by unbonded electrons can still be described effectively by the U and N processes, which also corroborates the rationality of estimating LTCs of crystalline solids by the perturbative phonon-phonon interactions. For the hierarchically bonded Cu_3_SbSe_3_, the experimental LTC data^21^ (Fig. 5(c)) demonstrate a nearly-temperature-independent nature, which significantly deviates from the classical *T*^−1^ relationship. A resonant-like phonon scattering rate^8^ 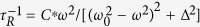 (rattling-like frequency *ω*_0_ = 1.0  THz, effective width *Δ* = 0.2 THz, and rattling-concentration-related parameter C^*^), which approximately describes the rattling-like thermal damping in the PCPL Cu_3_SbSe_3_, should be considered in addition to the other phonon scattering processes, including perturbative phonon-phonon U and N processes. The calculated LTC (Fig. 5(c)) accordingly displays an excellent agreement with the experiment in a wide temperature range.

## Summary

In summary, the three Cu-Sb-Se compounds exhibit significantly different lattice dynamic behaviors due to the different chemical-bond environments. Cu_3_SbSe_4_ and CuSbSe_2_ compounds are both in the crystalline state due to the homogeneous bonding strength, while the Cu_3_SbSe_3_ compound with chemical-bond hierarchy is in the part-crystalline part-liquid hybrid state at elevated temperatures. Harmonic and anharmonic properties vary with respect to the different crystal structures among Cu_3_SbSe_4_, CuSbSe_2_ and Cu_3_SbSe_3_ at low temperatures. For the close-packed diamond-like Cu_3_SbSe_4_, the phonon anharmonic interactions mainly originate from the Se atoms in the frameworks. While for CuSbSe_2_, the Sb^3+^ ions with lone pair *s*^2^ electrons dominate the anharmonicity by introducing an extra electrostatic repulsion. The weakly bonded Cu atoms in Cu_3_SbSe_3_, especially the Cu1 atoms, have a great influence on phonon-phonon anharmonic processes. For the description of thermal transport, traditional perturbative phonon-phonon interactions well depict LTCs of crystalline bulks (Cu_3_SbSe_4_ and CuSbSe_2_), whereas a rattling-like effective approach should be adopted in addition to the total scattering rate for the PCPL Cu_3_SbSe_3_ compound. Our analyses reveal the diverse lattice dynamics in the crystalline and PCPL Cu-Sb-Se materials, which may inspire additional approaches to designing materials with low LTCs for high-performance TE conversion.

## Methods

The first-principles calculations were performed in the framework of the density-function theory using the plane wave basis VASP code^32^,^33^, implementing the generalized gradient approximation (GGA) of Perdew-Burke-Ernzerhof (PBE) form^34^. The interactions between the ions and electrons were described by the all-electron projector augmented wave (PAW) method^35^,^36^, with plane waves up to a cutoff energy of 600 eV. The atomic configurations 3*d*^10^4*s*^1^ for Cu, 5*s*^2^5*p*^3^ for Sb, and 4*s*^2^4*p*^4^ for Se atoms were treated as the valence electrons. The Brillouin-zone integrations were performed on the grid of Monkhorst-Pack procedure^37^. For the unit cell of Cu_3_SbSe_3_, CuSbSe_2_, and Cu_3_SbSe_4_, 4 × 3 × 4, 4 × 6 × 2, and 5 × 5 × 3 *k*-point meshes were used, respectively. To calculate the phonon dispersion curves, we used the direct *ab initio* force-constant approach, which is implemented in the PHONON software by Parlinski^23^,^38^. Supercells with dimensions of 2 × 1 × 2, 2 × 3 × 1, and 2 × 2 × 1 were used for Cu_3_SbSe_3_, CuSbSe_2_, and Cu_3_SbSe_4_, respectively. High-symmetry points in Brillouin zones (*Γ* (0, 0, 0), *Z* (0.5, 0.5, −0.5), *N* (0.5, 0, 0), *P* (0.25, 0.25, 0.25), *X* (0, 0, 0.5) for Cu_3_SbSe_4_, *Γ* (0, 0, 0), *Z* (0, 0, 0.5), *X* (0.5, 0, 0), *U* (0.5, 0, 0.5), *Y* (0, 0.5, 0) for CuSbSe_2_, and *Γ* (0, 0, 0), *R* (0.5, 0.5, 0.5), *X* (0.5, 0, 0), *S* (0.5, 0.5, 0), *T* (0, 0.5, 0.5) for Cu_3_SbSe_3_) were considered in our phonon dispersion and Grüneisen parameter calculations. The ADP values are calculated based on the partial phonon density of states from DFT calculations. Molecular dynamics (MD) calculations were performed using the GGA of PBE form as implemented in the VASP code with the NVT ensemble. The PAW method was adopted, and supercells with 112 atoms (16.20 Å × 10.67 Å × 13.87 Å), 192 atoms (19.40 Å × 16.18 Å × 15.05 Å), and 64 atoms (11.47 Å × 11.47 Å × 11.41 Å) were used for Cu_3_SbSe_3_, CuSbSe_2_, and Cu_3_SbSe_4_, respectively.

To calculate the LTC in nonlinear phonon scattering, the Debye-Callaway model^39^ modified by Asen-Palmer *et al.*^40^ was applied, which can be expressed as


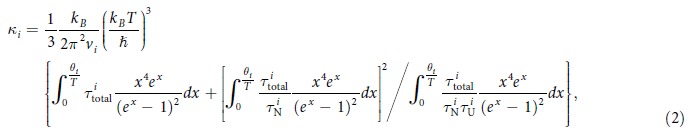


where *k*_*B*_, *v*, *θ*, and *τ* are the Boltzmann constant, phonon group velocity, Debye temperature and phonon relaxation time, respectively. Here *i* corresponds to the TA, TA’, or LA mode, while *x* takes the form as 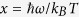. *U* and *N* stand for the phonon-phonon Umklapp and Normal processes, respectively, and further details of these expressions are included in Refs 8,40,41. In an ideal semiconductor, the scattering rates in the two processes are both considered to be proportional to *γ*^2^ according to traditional theories^41^,^42^,^43^, with *γ* being the Grüneisen parameter.

## Additional Information

**How to cite this article**: Qiu, W. *et al.* Diverse lattice dynamics in ternary Cu-Sb-Se compounds. *Sci. Rep.* **5**, 13643; doi: 10.1038/srep13643 (2015).

## Figures and Tables

**Table 1:** Debye temperature (*θ*), group velocities (*v*), and averaged Grüneisen parameters (*γ*) for the three compounds.

	Cu_3_SbSe_4_			CuSbSe_2_			Cu_3_SbSe_3_		
	TA	TA’	LA	TA	TA’	LA	TA	TA’	LA
*θ* (K)	64	67	77	48	56	59	39	40	45
*v* (m/s)	1806	2096	3859	1698	1786	3334	1568	1716	3272
*γ*	0.852	0.829	1.337	0.873	1.171	1.601	1.695	1.472	1.186
*γ*_ave_		1.006						1.451	

The average Grüneisen parameters are calculated by 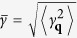, where 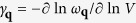, **q** and *V* are the wave vector and the equilibrium volume, respectively. The expended volume of 105% for the strained phonon calculations was used.
